# Bioethical issues in the family health strategy: Considerations for nursing care management

**DOI:** 10.1590/0034-7167-2022-0818

**Published:** 2024-08-19

**Authors:** Ieda Carla Almeida dos Santos de Souza Pastana, Gabriela Arantes Wagner, Geisa Colebrusco de Souza Gonçalves, Mariana Cabral Schveitzer

**Affiliations:** I Universidade Federal de São Paulo. São Paulo, São Paulo, Brazil

**Keywords:** Bioethics, Primary Health Care, Family Health, Nursing Care, Nursing, Bioética, Atención Primaria de Salud, Programa de Salud Familiar, Atención de Enfermería, Enfermería

## Abstract

**Objective:**

To understand the bioethical issues involved in the care management of nurses working in the Family Health Strategy.

**Method:**

A qualitative study was conducted through five focus groups with 36 nurses selected in the sample. Thematic content analysis was performed based on the bioethical framework, and the synthesis was presented in a conceptual map.

**Results:**

Bioethical issues were identified in the nursing care practice, related to both specific bioethical themes and the peculiarities of the work. Additionally, challenges and facilitators that interfere with addressing these issues were identified.

**Final considerations:**

Understanding the bioethical issues involved in the care management of nurses was possible with the theoretical support of different bioethical perspectives. The identified issues relate to persistent and current themes in the field of bioethics. However, some aspects intrinsic to daily practice are still imperceptible to professionals, contributing to the difficulty of discussing bioethics in this care model.

## RESUMEN

**Objetivo:**

Comprender cuestiones bioéticas involucradas en gestión del cuidado de enfermeros que actúan en Programa de Salud Familiar

**Método:**

Estudio cualitativo, realizado mediante cinco grupos focales con 36 enfermeros seleccionados en la muestra. Realizado análisis de contenido temático basado en referencial de la bioética y síntesis presentada en mapa conceptual.

**Resultados:**

Identificadas: cuestiones bioéticas presentes en la práctica de cuidado de enfermeros, relacionadas tanto a temáticas específicas de la bioética cuanto a particularidades laborales; así como dificultadores y facilitadores que interfieren en la conducción de esas cuestiones.

**Consideraciones finales:**

La comprensión de cuestiones bioéticas involucradas en gestión del cuidado de enfermeros fue posible con el apoyo teórico de diferentes corrientes bioéticas. Las cuestiones identificadas remeten a temáticas persistentes y actuales del campo de conocimiento de la bioética, mientras algunos contenidos intrínsecos al cotidiano todavía son imperceptibles para profesionales, contribuyendo para la dificultad de discutir bioética en ese modelo de atención.

## INTRODUCTION

One of the assumptions of the Family Health Strategy (ESF) is health care centered on the family, embedded in the physical, social, and cultural context. This requires professionals to have a broad understanding of the health-disease process, being an ideal setting for expanded clinical practice and continued care capable of enhancing actions that drive changes towards comprehensive care ^(1)^.

By employing predominantly soft and mild-hard care technologies for complex and varied demands, managing health care in the ESF considers criteria such as risk, vulnerability, resilience, with an ethical imperative that all health needs or suffering should be acknowledged. The individual and family care model aims to promote population care based on the principle of comprehensiveness^(2)^.

According to Cecílio ^(3)^, health care management is characterized by the use of technologies that aim to promote care coordination, user safety, and autonomy, considering their unique needs, based on six interdependent dimensions: individual; family; professional; organizational; systemic; and societal ^(3)^. In this study, the “organizational” and “professional” dimensions were explored with a focus on the ethical issues of nursing care practice in the ESF.

The literature points to the existence of relevant bioethical issues arising from care practice in the context of Primary Health Care, specifically in the ESF, understood under three primary perspectives, as highlighted by Zoboli and Fortes ^(4)^: ethical problems in relationships with users and families; in team relationships; and in relationships with the organization and the health system ^(4)^.

An integrative review supported these three perspectives on ethical issues and underscored that in the practice of nurses, ethical problems relate to difficulties in communication, autonomy, and respect in the relationship with users, situations in the relationship between professionals and academic training, challenges in defining responsibilities and specificities of each professional, unpreparedness to work in teams, difficulties in preserving the privacy of users due to structural deficiencies in health units, and an excess of families assigned to each team, leading to workload overload and little time for user care ^(5)^. These situations challenge professionals, even due to practical-theoretical difficulties, in proposing ethical solutions ^(6)^.

A Canadian study reaffirms the complexity of addressing bioethical issues in nurses’ clinical practice, emphasizing the need to improve ethical decision-making skills as a tool for professional practice ^(7)^. In addition, there is still a challenge represented by the scarcity of publications on ethics and bioethics in Primary Care compared to studies within the hospital setting ^(8)^.

Thus, identifying the bioethical issues involved in the organizational and professional care management of nurses working in the ESF, as well as the influencing aspects of the work process to address these situations, will allow us to understand this phenomenon and reflect on the best in service training practices for its management. Therefore, the question is: “What are the bioethical issues in the care management of nurses working in the Family Health Strategy from the perspective of these professionals, and what aspects influence them in addressing such issues?”

## OBJECTIVE

To understand the bioethical issues involved in the care management of nurses working in the Family Health Strategy.

## METHODS

### Ethical Aspects

This study adhered to national and international ethical guidelines following the norms of the National Health Council Resolution No. 466/2012. It was approved by the Research Ethics Committee of the Municipal Health Secretariat of São Paulo (SMS-SP), and the Federal University of São Paulo (UNIFESP).

The Informed Consent Form (ICF) was signed in duplicate by all participants and the researcher. To ensure the confidentiality and anonymity of the participants, an alphanumeric identification was employed, using the letter “N” for “nurse” and a cardinal numeral indicating the order of participation in each focus group (N1, N2...). These groups were identified with the letters “FG” and a cardinal numeral (FG1, FG2, FG3, FG4, and FG5).

### Theoretical-Methodological Framework

This is a descriptive qualitative study with data collection conducted through focus groups. It was based on the theoretical framework of bioethics and the methodological framework of qualitative health research ^(9)^. The COREQ protocol (Consolidated criteria for reporting qualitative research) was used to improve the presentation of results.

The material was analyzed based on the theoretical framework of the main currents of thought in bioethics that interface with care in Primary Healthcare: principlism, care ethics, protection ethics, intervention ethics, and the ethics of Moral Deliberation.

### Study Setting

From December 2019 to June 2020, the research was conducted in 36 basic health units (UBS) with ESF in the Eastern Zone of São Paulo, under the management contract of a Social Health Organization (OSS). Among the health services that make up this management contract, 51 are UBS with ESF, the setting for the participants in the research; of these, 36 services agreed to participate in the study. The coverage of ESF and Primary Care teams, at the time of data collection, in the eastern region of São Paulo, was 30.6% and 68.8%, respectively ^(10)^, indicating significant healthcare gaps.

### Participants

The participants in the study were technical managers nurse (TM) of UBS with ESF. According to the Federal Nursing Council ^(11)^, each UBS has only one TM nurse. The inclusion criteria were: being a nurse, being active at the time of data collection, and working as a TM in UBS with ESF. Those on leave were excluded. Fifty-one TM nurses met the inclusion criteria, of which 36 agreed to participate in the research.

### Data Collection

The focus groups took place by territory (with 6 to 12 participants), during working hours, previously arranged after participants’ acceptance. The number of participants followed the literature’s proposal, and the quantity and duration of the meetings were determined by the saturation criterion: they were concluded when the information started to become redundant and when the research’s established objectives were achieved ^(12)^.

The focus group consists of a collective interview, based on communication and interaction, aiming to gather detailed information on a subject, allowing an understanding of participants’ perceptions, beliefs, and attitudes. It is conducted by one or two moderators who guide the deepening of the proposed discussion on the study’s object ^(9,12)^.

Technical Health Supervision divided the focus groups, which were conducted in person and later remotely using Google Meet, due to the social distancing phase related to COVID-19. In both formats, those who agreed to participate in the research signed the ICF (from which they received a copy), answered the sociodemographic questionnaire, and authorized audio recording.

To guide the focus groups’ conduction, a script containing the following trigger questions was used: In your perception, are there bioethical issues experienced by nurses in the ESF? (Provide examples). In these situations, how did the team address the issue(s)? What were the consequences? What solution did the team find for the problem? What factors in the work process facilitated the management of these bioethical issues? What factors in the work process hindered the management of these bioethical issues?

The recordings were listened to and transcribed in full, preserving the fidelity of the information. The researcher, under the supervisor’s oversight, saved the files on hardware by following the National Research Ethics Committee (CONEP) Circular Letter No. 1, 2021 (CONEP, 2021). It is emphasized that all 36 research participants consented to the use of the data collected in the focus groups in its entirety. The average duration of each FG was 59 minutes, totaling 359 minutes of recording.

### Data Analysis

The exploration of the material was based on Bardin’s^(13)^ content analysis stages in combination with Minayo^(12)^. One researcher carried out the phases of pre-analysis, material exploration, treatment, inference, and interpretation of results, which were validated in consensus meetings with the other researchers. In the pre-analysis, initially, a floating reading was performed, followed by an exhaustive reading of the entire material, accompanied by active listening to the focus group recordings, aiming at data coding.

Data coding was performed by transforming textual data into cutting, aggregation, and enumeration, to represent the content, considering that the “appearance of a meaningful item or expression will be all the more significant the more this frequency is repeated” ^(13)^. This resulted in thematic categories, which were also validated by the researchers. For a better understanding of the results and synthesis, a conceptual map was created using the CmapTools 6.04 program.

## RESULTS

Five focus groups were conducted with the participation of 36 TM nurses from UBS with ESF, with an average of eight participants per group. Three occurred in person, and two remotely. FG1 happened in two sessions with the same participants. The formats did not present significant differences and provided an exploration of the subject with participation and exchange of experiences among the participants. In the two remote FGs, nurses, the researcher, the moderator, and the observer participated.

Regarding the sociodemographic profile, 92% were female, with an average age of 37 years. Most were married (81%), with children (72%), graduated between 2007 and 2017 (75%), and had post-graduation (specialization, 83%) or family health residency (17%). The professional experience of the majority was at least five years (64%).

In the focus groups, the researchers collected data from the transcription in its entirety and familiarized themselves with the content of the participants’ speeches to generate initial codes. These codes were then grouped into potential categories, which were refined and defined in relation to the research question. The defined categories and subcategories were systematically applied to the entire dataset, allowing the researchers to analyze patterns and connections. Finally, they organized the conceptual map and selected relevant excerpts.

**Figure 1:**
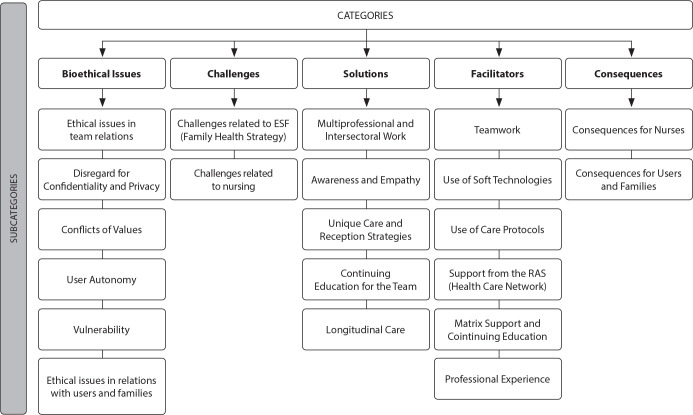
Distribution of categories and subcategories of focus groups, São Paulo, Brazil, 2021

Legend: The order of magnitude of the categories is represented by the size of the shapes and the font size of the categories, being directly proportional to the quantity of extracts for each category.

With the analysis of the focus groups, they obtained 237 excerpts, which were grouped into five categories for understanding the object: bioethical issues (73 excerpts - 31%), challenges (58 excerpts - 24%), solutions (49 excerpts - 21%), facilitators (29 excerpts - 12%), and consequences (28 excerpts - 12%). Each category was classified into subcategories related to the prevalent themes in the focus groups’ discourse (Figure 1).

### Bioethical Issues

This category encompassed 73 excerpts reflecting participants’ perceptions of bioethical issues arising from care practices in the ESF, grouped into six thematic subcategories: issues in team relations; disregard for confidentiality and privacy in the work context; conflicts of values; user autonomy; vulnerability; and issues in relations with users and families.

In the subcategory of “issues in team relations”, bioethical issues emerged from professional relationships in the ESF, perceived by participants as a lack of collaboration and commitment from some professionals, unpreparedness for work in the ESF context, disrespect in handling shared cases, and difficulties in defining professional roles, individual and shared responsibilities:

*So, to what extent is everyone committed and working together? To what extent is it important for me, for the CHW* [Community Health Worker]*, for the doctor, for another team?* (GF1; E6)

Concerning “disregard for confidentiality and privacy” in healthcare, participants reported difficulty when this principle jeopardizes other users:

*And not only that, in the tuberculosis issue for contacts, there’s also the issue of confidentiality. The patient has the right to keep their illness confidential. And from the moment I go in search of contacts, I lose that confidentiality...* (FG2; N2)

In the “conflicts of values” subcategory, different moral values coexisting in the work context were identified. These values stem from the socioeconomic and cultural diversity inherent in care practices:

*And another thing: what is right, what is wrong?* [...] *this is a very individual judgment.* [...] *When she mentioned income, it was, I think, her fifth pregnancy, she had two high-risk ones, had a miscarriage, various situations. I thought: “My God.” I asked: “Does your husband work?” She said: “No, just me.” The pregnancy was desired; I thought, I desired to say: “My God, what’s going on in your head?” But for her, she was smiling, happy...* (FG1; N9)

Regarding “user autonomy”, issues related to the professional’s scope of action on recommended therapeutic decisions and the user’s decision were found:

*...the case of a patient who also had TB* [tuberculosis] *and had just been diagnosed with HIV* [...] *and he didn’t accept home supervision because he didn’t want the community health worker at his house every day because neighbors would think: ‘Why is this professional at the neighbor’s house every day?’* [...] *So, he would go to the health unit, and he would lose track of time because he worked, didn’t have a fixed schedule* [...] *He had repeated many times that he would take the medication alone, that he didn’t need all that, that he didn’t want a food basket, that he just wanted to undergo his treatment, that he was responsible for himself. Even so, we tried to explain to him the importance of a health professional watching him take medication. And he would even say: ‘The tuberculosis treatment is six months, for HIV, I’ll take it my whole life. Are you going to come my whole life for me to take medicine? Why can I take HIV medication alone and not tuberculosis?’”* (FG2; N5)

In ethical matters, “vulnerability” was highlighted, especially in specific groups such as children and older people, and in situations involving violence and drug addiction in care:

*Violence is what we will bring the most* [...] *The protection network, especially for children, is so flawed, for the older person as well, that we want to take measures, but the part we need, support, we don’t have* [...] *it leaves us quite powerless as a strategy to deal with these cases.* (FG3; N2)

In “ethical issues arising from relations with users and families”, conflicts during the care interactions of the team with users and families were identified:

*For example, it took a long time to process a vasectomy or a DIU* [intrauterine device]*, then the woman comes to us and says, “See, I got pregnant, and it’s your fault because it took too long to put in my DIU.”* (FG4; N3)

### Challenges

This category consisted of 58 situations described in the focus groups, perceived as challenges, that is, factors that negatively interfere with the management of bioethical issues in the care practices of nurses TM in the ESF. The accounts were grouped into two subcategories of challenges: those related to the ESF’s work process, specifically difficulties in team effectiveness; and those linked to the nursing professional group, due to workload and the nurse’s centralization of responsibilities:

*And there are cases where I request urgency because if I’m asking, it’s because I want someone’s help to solve it together with me. And then, schedule it for a month later? No, I want it next week. And then, there is no space for us, they can’t come with me, “I’ll go with you, I want help.”* (FG3; N7)

*In addition to the pressure we have, it seems that everything is directed only at the nurse. Because sometimes, the pregnant woman went through another professional, and the other professional didn’t signal, and then it’s only for the nurse.* (FG1; N6)

### Solutions to Bioethical Issues

TM nurses reported 49 solutions to bioethical issues in their practice, involving the following themes: multiprofessional and intersectoral approach; development of awareness and empathy in the team; strategies that allow the uniqueness of care and reception; implementation of training and longitudinal care actions.

*In all cases, we see the similarity of teamwork, multiprofessional work* [...] *activating the SAE* [Specialized Care Service]*, activating the multiprofessional team with the NASF* [Family Health Support Center], [...] *articulating the entire network, to achieve the resolution of these bioethical issues.* (FG1; N3)

*I think, in our case, it was trying to involve other professionals, not just the nurse. Sometimes, the nurse, being the team leader, ends up centralizing a little more, but in these cases with these bioethical issues, then we try to involve other professionals for support* [...] (FG2; N1)

### Facilitators

This category portrayed participants’ perceptions of aspects related to the work process that facilitated the management of bioethical issues experienced. For the distribution of the 29 excerpts extracted, six subcategories were defined: 1) teamwork; 2) use of soft care technologies (bond, active listening); 3) mild-hard (care protocols, technical-scientific knowledge); 4) realization of actions in the Health Care Networks (RAS) and 5) strategies for continuing education and matrix support, and 6) professional experience.

*The facilitator we have in the Family Health Strategy is that we are in patients’ homes, so the bond is quite strong with them. So I’m there, I enter their homes, so they sometimes see us as a family member... we don’t just take care of physical health, we take care of everything.* (FG1; N3)

*...I think that technical-scientific knowledge is essential for us to know where to retrieve this support, be it in legislation, be it in the code of ethics. So, if you are technically empowered, you can, from a lot of situations, find resolutions.* (FG4; N1)

### Consequences

In total, 28 consequences of bioethical issues were reported, classified into two subcategories: consequences for users and/or families, such as loss of bond with the team or service, family crisis, failure in follow-up and non-adherence to treatment, as well as low case resolution; and consequences for professionals, such as insecurity, resilience development, illness, dissatisfaction, demotivation, and loss of credibility.

*...negative issues, they are very strong in this regard, which we carry, what we will carry for the rest of our lives, but I also firmly believe that it is in these situations that we develop resilience, and it is in these situations that we learn.* (FG1; N4)

*Really, I agree with N2. I think the loss of bond is the biggest consequence for us as a Basic Health Unit because it becomes much more difficult later to monitor the entire treatment, reverse this situation...* (FG5; N5)

**Figure 2:**
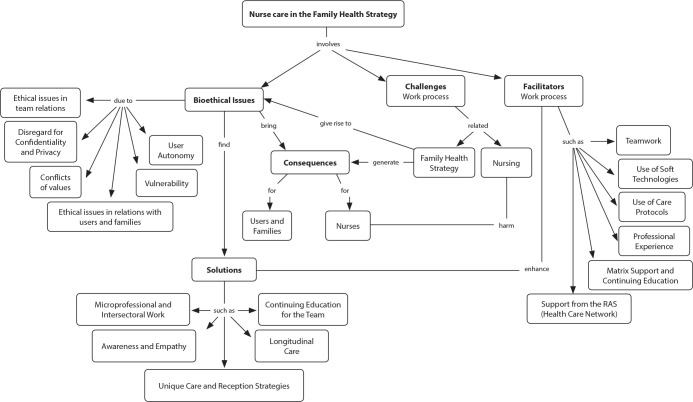
Conceptual Map on the Bioethical Issues of Nursing Care in the Family Health Strategy, São Paulo, Brazil, 2021

The results allowed for the construction of the conceptual map of bioethical issues present in the care practices of Family Health Strategy nurses, including solutions, facilitators, challenges, and consequences (Figure 2).

## DISCUSSION

In this research, it was identified that the ESF is permeated by bioethical issues that reflect the complexity of care in this context due to the intensity of relationships, teamwork, and the plurality of values involved in individual and family care. Such issues also represent diversity in aspects related to bioethics, as illustrated in the conceptual map.

Among the bioethical issues identified in the focus groups, those related to teamwork, confidentiality, and privacy, and those arising from relationships with users and families corroborate the findings of Zoboli and Fortes ^(4)^. In this study, they represented 58% of the extracts, demonstrating their relevance in the ESF’s practice. It is noteworthy that ethical problems arising from conflicting relationships within the team or between the team and users can be understood from the perspective of care ethics, which focuses on responsibility in human relationships.

In the care ethics concept of Gilligan, an ethical problem arises when there is a conflict of responsibility in human relationships, and the resolution of this conflict aims to ensure the maintenance of care relationships ^(14)^. From this perspective, the solution to ethical problems of this nature, according to Zoboli ^(15)^, involves activating the network of relationships through non-violent, cooperative, and non-competitive communication that promotes the inclusion of all to strengthen rather than break existing connections ^(15)^. This was precisely demonstrated in reports where bonding and therapeutic continuity were considered important by professionals.

Regarding bioethical issues related to user confidentiality and privacy, although present since principled ethics, the results showed that they still occur in the care practice of ESF nurses. This is an inherent aspect of nursing practice, provided for in the Code of Ethics for Nursing Professionals ^(11)^.

According to Junges and collaborators^(16)^, this legal approach to resolving issues of confidentiality and confidentiality in care practice proves insufficient to respond to the various situations required during the ESF’s longitudinal care work, which is characteristic of the model of care that proposes an approach of reception, trust, and bonding.

Participants considered that bioethical issues related to conflicts of values stemmed from socioeconomic and cultural diversity in the working context and configure themselves into conflicts when they contrast with the moral values of professionals. The literature shows that the occurrence of bioethical issues in healthcare often makes explicit cultural, value, and belief differences so that care actions can unfold in different interpretations of ways of life ^(17)^.

Regarding the challenges of bioethical problems, participants listed difficulties in the development of teamwork, essentially in its effectiveness, weaknesses in teamwork competence, and the orientation of work towards goal achievement and productivity. Interfering in the assistance offered to the community, difficulties related to teamwork in the ESF have been reported as an obstacle to integrated work: lack of cooperation between professionals, rigidly hierarchical organizational structures, technical and social inequality, high turnover, insufficient professional quantity, and low qualification ^(18)^.

The multiprofessional nature of the work of ESF reveals an indispensable need and, at the same time, a challenge regarding the transition from the configuration of grouping people to the constitution of a team, characterized by interaction and integration among professionals that result in the formation of a common project ^(19)^. Furthermore, although not emphasized by professionals in the FGs, the strategy of moral deliberation-similar to teamwork-as a possibility of resolving bioethical problems requires integrated action and recognizes the limitation of individual care and decision-making ^(17)^.

Reaffirming the literature, the results from focus groups also showed that service management based essentially on outcome indicators was a hindrance: it is one of the main factors that cuts across the team’s work process and results in damages in handling bioethical issues ^(19)^. In other words, in the focus groups, TM nurses recognized in the quality of bioethical problems the fact that productivity goals do not always translate into values such as access, quality, and safety of care.

Furthermore, elements of the daily work that would require actions from service management reveal worker fatigue: workload, excessive demand, accumulation of functions beyond care, complexity of health demands, and difficulties in meeting user expectations. This negatively impacts access, comprehensiveness, quality, and safety of care ^(20-21)^.

Even with the presented difficulties, participants pointed out various decision-making strategies grounded in the values of the principles and guidelines of this care model as solutions to address bioethical issues. In this regard, the importance of considering the polysemic theme of comprehensive care, both “focused comprehensiveness” resulting from the integration of various knowledge in multiprofessional care and “expanded comprehensiveness” derived from the articulation of each service in the Health Care Network (RAS) and intersectoral institutions is observed ^(22)^.

Other solutions proposed for bioethical issues were “awareness and empathy” and “team training.” The former begins with problematizing these issues, aiming to ensure the constitutional right to health and humanized care. Regarding team training, to bring about changes in care practices, they emphasized the need to establish a dialogue on the work process by problematizing it ^(23-24)^.

The solutions proposed in the focus groups align with care ethics proposals, as they presuppose practical results that require non-coercive dialogical relationships and change through awareness among those involved in the bioethical issue. In this context, moral deliberation can stand out not only as a method but also as a continuous process of learning applied ethics, based on the collective construction of practical solutions for bioethical problems, characterized by the continuous exercise of investigating intermediate and prudent courses of action ^(17,25)^.

Participants acknowledged team decision-making and the development of unique therapeutic projects contributed to a more assertive and prudent solution to identified bioethical issues. Such choices are related to intermediate courses of action and a problematizing approach. Additionally, by sharing the case and decision, there is also the outline of a common responsibility for the team and, consequently, a decrease in workload and the suffering that may result from these decisions.

The facilitators’ category encompassed teamwork, the use of soft and mild-hard care technologies, support from the RAS, continuing education, matrix support, and professional experience in PHC. “Teamwork” was perceived as a facilitator when present and a hindrance when absent. When teamwork is effective in the work process, it was considered a powerful facilitator for handling bioethical issues.

Additionally, there was a recognition of the importance of bonding and qualified listening as propellers for comprehensive and humanized care, serving as facilitators in addressing bioethical issues in the ESF to understand the expanded concept of health. Bonding, as a soft technology in relationships within the ESF, is embedded in the basic guidelines of this care model, establishing responsibility for the assigned area of the territory and, consequently, the need for an interaction that generates bonds-longitudinal, humanized, and integral-between health workers and users ^(26)^.

The support of the Health Care Network and continuing education was also present, serving as both facilitators and solutions to manage bioethical issues. As a strategy for reorganizing the health system, the ESF implies being recognized as the coordinator of care and a central element in communication with the RAS. The organization of health services into networks presupposes a shift from fragmented systems and is realized through high-quality Primary Care, with teams capable of expanding interprofessional action beyond the local scope, involving other teams from different points in the network that collaborate with users and the community to achieve comprehensive care^(19)^.

In this sense, the focus group reports highlighted the relevance of matrix support provided by the multiprofessional team. For them, it is a potent in-service continuing education action that contributes to expanding the nurse’s clinical skills and to transcending the biomedical model. The continuing education process for the bioethics theme includes the problematization of situations experienced by ESF teams and proves to be a powerful qualification strategy to identify and propose solutions to ethical problems, incorporating concepts, theories, and methods of bioethics ^(27)^.

Among the main consequences of bioethical issues mentioned by focus group participants, “consequences for ESF nurses” and “consequences for users and family” stood out. For nurses, positive aspects were included (e.g., the development of resilience for coping); however, they considered most consequences negative (insecurity, illness, dissatisfaction, discouragement, and loss of credibility).

A study showed that bioethical issues in the context of Primary Care affect workers, users, managers, and the structure of health work itself. Thus, it demonstrated the need for ESF professionals to foster discussions to improve communication and teamwork, as well as enhance fundamental skills for the interdisciplinary and expanded practice that ethics in health care presupposes ^(28)^.

In this study, the loss of bonding and family crisis were identified as consequences for users and families in managing the occurrence of bioethical issues that violated the user’s confidentiality and privacy, whether in a dilemmatic approach or from a legal, deontological perspective. The literature also points to the team’s discredit resulting from a breach of trust, while indicating that communication is key to its solution ^(29)^.

The legal perspective focuses on duties expressed in the professional code, sometimes insufficient for the everyday bioethical issues of the ESF, which presuppose practices such as reception, trust, and bonding. These are necessary for the agreement to share information between users, family, and professionals, considering the well-being, user rights, and intersubjectivity in discussing problems ^(16)^. Indeed, in the ESF’s performance, very specific bioethical issues are evident, which should be overcome through a dialogue-driven construction with the participation of all ^(24)^.

### Study Limitations

The limitations of this study correspond to the need to include the perceptions of other actors regarding bioethical issues in the nursing care practice of ESF, such as users and health service managers, to complement the findings. It is worth noting that due to the epidemiological scenario related to the COVID-19 pandemic, it was necessary to change the data collection format from in person to virtual.

### Contributions to Nursing and Public Health

This study contributes with essential elements: reflection on the bioethical issues inherent in the care practice and the work process of nurses in the ESF, and recognition of the importance of using the assumptions of moral deliberation for the analysis and resolution of these issues.

## FINAL CONSIDERATIONS

The bioethical issues in this study referred to persistent themes in the field of bioethics. These issues pose a challenge as they present various possible courses of action, demanding careful consideration to choose the best alternative.

The understanding of bioethical issues involved in the care management of nurses working in the Family Health Strategy was possible by different bioethical currents as a theoretical framework. In this study, there were observed approximations with principlism, care ethics, and interface currents of collective health: bioethics of protection and intervention. It is emphasized that to understand the complexity of bioethical issues in this care context, the complementarity of different currents becomes necessary.

There were more challenges than facilitators in addressing bioethical issues, indicating the need to reorganize the work process of teams to enable the identification, analysis, and proposition of solutions to these issues.

Solutions to bioethical issues were related to collective decisions, reinforcing the importance of interprofessional and interdisciplinary teams in investigating bioethical issues to achieve intermediate courses of action. Such solutions are achieved when there is longitudinal care, continuing education, and a systematic approach proposed by moral deliberation.

The facilitators identified in this research strongly converged with solutions to bioethical issues, considering the quality of care and preventing deleterious consequences for professionals and users, family, and the community.

Implications and recommendations for the care practice of bioethical issues include: promoting continuing education with the support of problematizing learning methodologies; valuing the uniqueness of care, using soft technologies; providing spaces for discussion, structured approach such as the methodology of moral deliberation to make decisions; investing in strategies that strengthen teamwork and contribute to resolving needs, aiming to enhance principles and guidelines of the Unified Health System (SUS); developing qualitative care indicators that recognize relational aspects of actions performed in the ESF, such as humanization, empathy, bonding, reception, respect for autonomy, expanded clinic, and user satisfaction in care outcomes.

Implications for future research include conducting studies that understand the perceptions of other actors, users, and managers, to expand knowledge about bioethical issues and their approaches in care management in the ESF, with a focus on methodologies that encompass problematization, such as focus groups and action research.
