# Supplementary material for: Bioethical issues in the family health strategy: Considerations for nursing care management
Source: Rev Bras Enferm. 2024 Aug 19;77(Suppl 4):e20220818. doi: 10.1590/0034-7167-2022-0818 (PMC11338529; doi:10.1590/0034-7167-2022-0818)
Supplement: Translation

# Translation: Questões bioéticas na estratégia saúde da família: considerações para a gestão do cuidado de enfermagem

**DOI:** 10.1590/0034-7167-2022-0818pt

**Authors:** Ieda Carla Almeida dos Santos de Souza Pastana, Gabriela Arantes Wagner, Geisa Colebrusco de Souza Gonçalves, Mariana Cabral Schveitzer

**Affiliations:** I Universidade Federal de São Paulo. São Paulo, São Paulo, Brasil

**Keywords:** Bioética, Atenção Primária à, Saúde, Estratégia Saúde da Família, Cuidados de Enfermagem, Enfermagem

## Abstract

**Objetivo:**

Compreender as questões bioéticas envolvidas na gestão do cuidado dos enfermeiros que atuam na Estratégia Saúde da Família

**Método:**

Estudo qualitativo, realizado por meio de cinco grupos focais com 36 enfermeiros selecionados na amostra. Efetuou-se análise de conteúdo temática com base no referencial da bioética e síntese apresentada em mapa conceitual.

**Resultados:**

Identificaram-se: questões bioéticas presentes na prática de cuidado dos enfermeiros, relacionadas tanto às temáticas específicas da bioética quanto às particularidades do trabalho; e dificultadores e facilitadores que interferem na condução dessas questões.

**Considerações finais:**

A compreensão das questões bioéticas envolvidas na gestão do cuidado dos enfermeiros foi possível com o apoio teórico de diferentes correntes bioéticas. As questões identificadas remetem a temáticas persistentes e atuais do campo de conhecimento da bioética, entretanto alguns conteúdos intrínsecos ao cotidiano ainda são imperceptíveis para os profissionais, contribuindo para a dificuldade de discutir bioética nesse modelo de atenção.

## INTRODUÇÃO

Um dos pressupostos da Estratégia de Saúde da Família (ESF) é a atenção à saúde centrada na família, inserida no contexto físico, social e cultural. Isso requer dos profissionais compreensão ampliada do processo saúde-doença, sendo cenário privilegiado para prática da clínica ampliada, do cuidado continuado, capaz de potencializar ações que impulsionam mudanças voltadas à construção da integralidade ^(1)^.

Ao utilizar tecnologias de cuidado majoritariamente leve e leve-duras para as demandas complexas e variadas, no manejo das necessidades de saúde no território de abrangência, a ESF considera critérios de risco, vulnerabilidade, resiliência, com imperativo ético de que toda necessidade de saúde ou sofrimento devam ser acolhidos. O modelo de cuidado individual e familiar busca promover o cuidado da população com base no princípio da integralidade ^(2)^.

Segundo Cecílio^(3)^, a gestão do cuidado em saúde caracteriza-se pela utilização de tecnologias que visam promover coordenação do cuidado, segurança e autonomia do usuário, considerando suas necessidades singulares, com base em seis dimensões interdependentes: individual; familiar; profissional; organizacional; sistêmica; e societária ^(3)^. Neste estudo, foram exploradas as dimensões “organizacional” e “profissional” com enfoque nas questões éticas da prática do cuidado dos enfermeiros que atuam na ESF.

A literatura aponta a existência de relevantes problemas bioéticos, que emergem da prática de cuidado no contexto da Atenção Primária à Saúde, especificamente na ESF, e, de acordo com Zoboli e Fortes^(4)^, entendidos sob três perspectivas primordiais: problemas éticos nas relações com usuários e família; nas relações da equipe; e nas relações com a organização e com o sistema de saúde ^(4)^.

Revisão integrativa corroborou essas três perspectivas de problemas éticos e destacou que, na prática dos enfermeiros, os problemas éticos referem-se às dificuldades na comunicação, autonomia e respeito na relação com os usuários, situações da relação entre os profissionais e formação acadêmica, desafio em delimitar responsabilidades e especificidades de cada profissional, despreparo para trabalhar em equipe, dificuldades em preservar a privacidade dos usuários por deficiências estruturais das unidades de saúde e excesso de famílias adscritas para cada equipe, que implica sobrecarga de trabalho e pouco tempo para o cuidado aos usuários ^(5)^. Tais situações desafiam os profissionais, até mesmo pelas dificuldades prático-teóricas, na proposição de soluções éticas vivenciadas ^(6)^.

Estudo canadense reafirma a complexidade de abordar questões bioéticas na prática clínica dos enfermeiros, destacando a necessidade de aprimoramento da habilidade de tomada de decisão ética como ferramenta para a prática profissional ^(7)^. Além disso, ainda há uma dificuldade representada pela escassez de publicações sobre ética e bioética na Atenção Primária, em comparação com estudos dentro do cenário hospitalar ^(8)^.

Assim, identificar as questões bioéticas envolvidas na gestão do cuidado organizacional e profissional dos enfermeiros atuantes na ESF, bem como os aspectos influenciadores do processo de trabalho para o enfrentamento dessas situações, permitirá compreender esse fenômeno e refletir sobre as melhores práticas de formação em serviço para a sua condução. Dessa forma, pergunta-se: “Quais são as questões bioéticas na gestão do cuidado dos enfermeiros atuantes na Estratégia Saúde da Família sob a perspectiva desses profissionais e quais aspectos os influenciam na condução de tais questões?”

## OBJETIVO

Compreender as questões bioéticas envolvidas na gestão do cuidado dos enfermeiros que atuam na Estratégia Saúde da Família (ESF).

## MÉTODOS

### Aspectos Éticos

Este estudo obedeceu às diretrizes de ética nacionais e internacionais, seguindo as normas da Resolução do Conselho Nacional de Saúde nº 466 de 2012 e foi aprovado pelo Comitê de Ética em Pesquisa da Secretaria Municipal de Saúde de São Paulo (SMS-SP) e da Universidade Federal de São Paulo (UNIFESP).

O Termo de Consentimento Livre e Esclarecido (TCLE) foi assinado em duas vias por todos os participantes e pela pesquisadora. Para garantia do sigilo e anonimato dos participantes, foi empregada identificação alfanumérica, com uso da letra “E” de “enfermeiro” e um numeral cardinal indicando a ordem de participação de cada um nos grupos focais (E1, E2…). Esses grupos foram identificados com as letras “GF” e um numeral cardinal (GF1, GF2, GF3, GF4 e GF5).

### Referencial teórico-metodológico

Estudo qualitativo descritivo cuja coleta de dados foi realizada por meio de grupos focais. Fundamentou-se no referencial teórico da bioética e referencial metodológico da pesquisa qualitativa em saúde^(9)^. Foi utilizado o protocolo COREQ (*Consolidated criteria for reporting qualitative research*) para aperfeiçoar a apresentação dos resultados.

A análise do material foi realizada por meio do referencial teórico das principais correntes de pensamento da bioética que têm interface com o cuidado na Atenção Primária à Saúde: principialismo, bioética do cuidado, da proteção, de intervenção e a bioética da Deliberação Moral.

### Cenário de Estudo

No período de dezembro de 2019 a junho de 2020, realizou-se a pesquisa em 36 unidades básicas de saúde com ESF, da Zona Leste da cidade de São Paulo, sob contrato de gestão de uma Organização Social de Saúde (OSS). Dentre os serviços de saúde que compõem esse contrato de gestão, 51 são UBS com ESF, cenário de atuação dos participantes da pesquisa; destes, 36 serviços aceitaram participar da pesquisa. A cobertura de equipes de ESF e de Atenção Primária, no momento de coleta de dados, na região leste do município de São Paulo, era de 30,6% e 68,8%, respectivamente^(10)^, indicando vazios assistenciais significativos.

### Participantes da Pesquisa

Os participantes da pesquisa foram enfermeiros responsáveis técnicos (RTs) das UBS com ESF. De acordo com o Conselho Federal de Enfermagem ^(11)^, cada UBS conta com apenas um enfermeiro RT. Os critérios de inclusão foram: ser enfermeiro, em atividade no momento de coleta de dados, atuante como RT em UBS com ESF. Foram excluídos aqueles que estavam em afastamento. Atenderam aos critérios de inclusão 51 enfermeiros RTs, dos quais 36 concordaram em participar da pesquisa.

### Coleta de Dados

Os grupos focais ocorreram por território (de 6 a 12 participantes), no horário de trabalho, previamente combinado após aceitação dos participantes. O número de participantes seguiu o proposto pela literatura; e a quantidade e duração dos encontros foram determinados pelo critério de saturação: foram encerrados quando as informações começaram a ficar redundantes e quando os objetivos estabelecidos na pesquisa foram alcançados ^(12)^.

O grupo focal consiste em entrevista coletiva, baseada na comunicação e interação, com objetivo de reunir informações detalhadas sobre um tema, permitindo a compreensão de percepções, crenças e atitudes dos participantes; é conduzido por um ou dois moderadores, que direcionam o aprofundamento da discussão proposta sobre o objeto de estudo^(9,12)^.

Os grupos focais foram divididos por Supervisão Técnica de Saúde e foram realizados de forma presencial e posteriormente de forma remota com uso do Google Meet, por causa da fase de distanciamento social relacionada à covid-19. Em ambos os formatos, os que aceitaram participar da pesquisa assinaram o TCLE (do qual receberam uma cópia), responderam ao questionário sociodemográfico e autorizaram a gravação em áudio.

Para nortear a condução dos grupos focais, foi usado um roteiro contendo as seguintes perguntas disparadoras: Na percepção de vocês, há questões bioéticas vivenciadas pelos enfermeiros na ESF? (Exemplifique). Nessas situações, como a equipe abordou a(s) questão(ões)? Quais foram suas consequências? Qual foi a solução encontrada pela equipe para o problema? Quais fatores do processo de trabalho facilitaram a condução dessas questões bioéticas? Quais fatores do processo de trabalho dificultaram a condução dessas questões bioéticas?

As gravações foram ouvidas e transcritas na íntegra preservando a fidedignidade das informações. Os arquivos foram salvos em hardware, pela pesquisadora, sob a supervisão da pesquisadora-orientadora, conforme Carta Circular do Conselho Nacional de Ética em Pesquisa (CONEP) nº 1, de 2021 (CONEP, 2021). Destaca-se que os 36 participantes da pesquisa consentiram a utilização dos dados coletados nos grupos focais em sua totalidade. A duração média de cada GF foi de 59 minutos, totalizando 359 minutos de gravação.

### Análise de Dados

A exploração do material deu-se com base nas etapas de análise de conteúdo de Bardin ^(13)^ em combinação com Minayo ^(12)^. Procedeu-se às fases de pré-análise, exploração do material, tratamento, inferência e interpretação dos resultados, realizadas por uma pesquisadora e validadas em reuniões de consenso com as demais pesquisadoras. Na pré-análise, foi efetuada, inicialmente, leitura flutuante, seguida da leitura exaustiva de todo o material, acompanhada pela escuta ativa das gravações dos GFs, com a finalidade de codificação dos dados.

A codificação dos dados deu-se por transformação de dados textuais em recorte, agregação e enumeração, para representação do conteúdo, considerando que a “aparição de item de sentido ou de expressão será tanto mais significativa quanto mais essa frequência se repetir” ^(13)^. Isso resultou em categorias temáticas, que também foram validadas pelas pesquisadoras. Para melhor compreensão dos resultados e síntese, construiu-se um mapa conceitual no programa CmapTools 6,04.

## RESULTADOS

Foram realizados cinco grupos focais com participação de 36 enfermeiros RTs das UBS com ESF, com oito participantes em média por grupo. Três ocorreram presencialmente; e dois, remotamente. O GF1 ocorreu em dois momentos, com os mesmos participantes. Os formatos não apresentaram diferenças expressivas e propiciaram exploração do objeto com participação e troca de experiências entre os participantes. Nos dois GFs remotos, participaram os enfermeiros, pesquisadora, moderador e observador.

Quanto ao perfil sociodemográfico, 92% eram do sexo feminino, com média de idade de 37 anos. A maioria era casada (81%), com filhos (72%), formou-se entre 2007 e 2017 (75%) e tinha pós-graduação (especialização, 83%) ou residência em saúde da família (17%). O tempo de atuação profissional da maioria era de no mínimo cinco anos (64%).

Nos GFs, os pesquisadores coletaram os dados da transcrição em sua totalidade e familiarizaram-se com o conteúdo das falas dos participantes para gerar os códigos iniciais. Esses códigos foram então agrupados em categorias potenciais, que foram refinadas e definidas em relação à questão de pesquisa. As categorias e subcategorias definidas foram sistematicamente aplicadas a todo o conjunto de dados, permitindo que os pesquisadores analisassem padrões e conexões. Finalmente, eles organizaram o mapa conceitual e selecionaram extratos relevantes.

Com a análise dos GFs, obtiveram 237 extratos, que foram agrupados em cinco categorias para compreensão do objeto: questões bioéticas (73 extratos - 31%), dificultadores (58 extratos - 24%), soluções (49 extratos - 21%), facilitadores (29 extratos - 12%) e consequências (28 extratos - 12%). Cada categoria foi classificada em subcategorias, relacionadas às temáticas prevalentes nos discursos dos grupos focais (Figura 1).

**Figura 1:** Distribuição das categorias e subcategorias dos grupos focais, São Paulo, Brasil, 2021

Legenda: A ordem de grandeza das categorias está representada pelo tamanho das formas e pelo tamanho da fonte das categorias, sendo diretamente proporcional à quantidade de extratos de cada categoria.

### Questões Bioéticas

Esta categoria abrangeu 73 extratos das percepções dos participantes sobre questões bioéticas provenientes da prática de cuidado na ESF, agrupadas em seis subcategorias temáticas: problemas na relação da equipe; desrespeito ao sigilo e à privacidade no contexto de atuação; conflitos de valores; autonomia do usuário; vulnerabilidade; e problemas nas relações com usuários e famílias.

Na subcategoria “problemas na relação com a equipe”, as questões bioéticas emergiram das relações profissionais na ESF, percebidas pelos participantes como falta de colaboração e compromisso de alguns profissionais, despreparo para o trabalho no contexto da ESF, desrespeito no tratamento de casos compartilhados e dificuldades para delimitar atuação profissional, especificidades e responsabilidades individuais e compartilhadas:

*Então até que ponto todo mundo está comprometido e está trabalhando junto? Até que ponto é importante para mim, é importante para o ACS, é importante para o médico, é importante para outra equipe?* (GF1; E6)

Quanto ao “desrespeito ao sigilo e à privacidade” no cuidado em saúde do usuário, os participantes relataram dificuldade quando esse princípio coloca em risco outros usuários:

*E não só isso, na questão da tuberculose para os contatos, existe também a questão do sigilo. O paciente tem o direito de ter o sigilo sobre a sua doença. E a partir do momento que eu vou na busca dos contatos, eu deixo de ter esse sigilo* [...] (GF2; E2)

Em “conflito de valores”, identificaram-se diferentes valores morais que coexistem no contexto de atuação. Eles são fruto da diversidade socioeconômica e cultural que perpassa a prática do cuidado:

*E outra coisa: o que é o certo, o que é o errado?* […] *esse é um julgamento que é muito individual.* […] *Quando ela falou a renda, já era acho que a quinta gestação dela, ela teve duas de alto risco, já tinha aborto, várias situações. Eu falei: “Meu Deus.” Eu falei: “Seu esposo trabalha?”. Ela falou assim: “Não, só eu.” É desejada, era desejada a gestação, eu pensava, a minha vontade era falar: “Meu Deus, o que está passando na sua cabeça?” Mas, para ela, ela estava sorridente, feliz* […] (GF1; E9)

Quanto à “autonomia do usuário”, foram encontradas questões relacionadas ao limite de atuação do profissional sobre as decisões terapêuticas recomendadas e decisão do usuário:

[…] *o caso de um paciente que também era TB* [tuberculose] *que ele tinha acabado de descobrir o diagnóstico de HIV* […] *e ele não aceitava ser supervisionado em domicílio, porque ele não queria o agente comunitário todos os dias na casa dele porque os vizinhos iam pensar: “Por que esse profissional está todo dia na casa do vizinho?”* […] *Aí ele ia para a unidade e ele se perdia com o horário porque ele trabalhava, não tinha horário fixo* […] *Ele já tinha repetido várias vezes que ele faria o uso da medicação sozinho, que ele não precisava de tudo aquilo, que ele não queria cesta básica, que ele só queria fazer o tratamento dele, que ele era responsável por si. Mesmo assim, a gente tentava explicar para ele a importância de um profissional da saúde vendo ele tomar medicação. E ele até falava: “O tratamento da tuberculose é seis meses, do HIV eu vou tomar a vida inteira, você vai vir a vida inteira para eu tomar remédio? Por que o do HIV eu posso tomar sozinho e da tuberculose não?* (GF2; E5)

Nas questões éticas, foram destacadas a “vulnerabilidade”, principalmente de grupos específicos, como crianças e idosos, e em situações envolvendo violência e drogadição no cuidado:

*A violência é o que mais a gente vai trazer* […] *A rede de proteção, principalmente a criança, é tão falha, do idoso também, que a gente quer tomar condutas, e a parte que a gente precisa, do apoio, a gente não tem* […] *“deixa a gente bastante impotente enquanto estratégia para lidar com esses casos.* (GF3; E2)

Na subcategoria “problemas éticos nas relações com usuários e famílias”, foram identificadas questões de conflito durante as interações de cuidado da equipe com os usuários e famílias:

*Então, por exemplo, demorou para um processo de fazer uma vasectomia ou um DIU* [dispositivo intrauterino]*, aí a mulher vem até nós e fala: “Está vendo, engravidei, e a culpa foi sua porque demorou para colocar o meu DIU.”* (GF4; E3)

### Dificultadores

Esta categoria foi composta por 58 situações descritas nos grupos focais, percebidas como dificultadores, ou seja, aquilo que interfere negativamente na condução das questões bioéticas presentes na prática de cuidado dos enfermeiros RTs da ESF. Os relatos foram agrupados em duas subcategorias de dificultadores: os relacionados ao processo de trabalho da ESF, especificamente dificuldades de efetivação do trabalho em equipe; e aqueles ligados ao grupo profissional da enfermagem, pela sobrecarga de trabalho e centralização de responsabilidades do enfermeiro:

*E aí tem casos que eu peço urgência, porque se eu estou pedindo, é porque eu quero ajuda de alguém para resolver junto comigo. E aí marca para depois de um mês? Não, eu quero semana que vem. E aí não abre esse espaço para a gente, não pode abrir para ir comigo, “eu vou junto, eu quero ajuda.”* (GF3; E7)

*Além da cobrança que tem, parece que é voltado tudo só para o enfermeiro. Porque, às vezes, a gestante passou por um outro profissional, que o outro profissional não sinalizou, e aí fica só para o enfermeiro*. (GF1; E6)

### Soluções diante de questões bioéticas

Os enfermeiros RTs relataram 49 soluções para questões bioéticas presentes na atuação desses profissionais, envolvendo as seguintes temáticas: abordagem multiprofissional e intersetorial; desenvolvimento da conscientização e empatia na equipe; estratégias que permitem a singularidade do cuidado e do acolhimento; implementação de ações de capacitação e cuidado longitudinal.

*Em todos os casos, nós vemos a semelhança do trabalho em equipe, do trabalho multiprofissional* […] *acionar o SAE, acionar a equipe multi com o NASF,* […] *de articular toda a rede, para a gente conseguir a conclusão desses assuntos bioéticos.* (GF1; E3)

*Acho que, no nosso caso, foi tentar envolver outros profissionais, não somente o enfermeiro. Às vezes, o enfermeiro, por ser o líder da equipe, ele acaba centralizando um pouco mais, mas nesses casos que têm essas questões bioéticas, então a gente tenta envolver os outros profissionais para ter um apoio* […] (GF2; E1)

### Facilitadores

Esta categoria retratou as percepções dos participantes sobre os aspectos relacionados ao processo de trabalho que facilitaram a condução das questões bioéticas vivenciadas. Para a distribuição dos 29 excertos extraídos, foram definidas seis subcategorias: 1) trabalho em equipe; 2) uso das tecnologias do cuidado leves (vínculo, escuta ativa); 3) leve-duras (protocolos assistenciais, conhecimento técnico-científico); 4) efetivação das ações das redes de atenção à saúde (RAS) e das 5) estratégias de educação permanente e matriciamento, e 6) experiência profissional.

*O facilitador que a gente tem na Estratégia Saúde da Família é que a gente está na casa dos pacientes, então o vínculo é bastante forte com eles. Então eu estou lá, eu entro na casa, então eles nos veem às vezes como um membro da família… a gente não cuida só de saúde física, a gente cuida de tudo.* (GF1; E 3)

[…] *eu acho que o conhecimento técnico-científico, ele é fundamental até para a gente poder saber onde resgatar esse respaldo, seja na legislação, seja no código de ética. Então, se você está tecnicamente empoderado, você consegue, a partir de N situações, achar as resoluções.”* (GF4; E 1)

### Consequências

Foram relatadas 28 consequências das questões bioéticas, classificadas em duas subcategorias: consequências para usuários e/ou famílias, tais como perda do vínculo com equipe ou serviço, crise familiar, falha no acompanhamento e não adesão ao tratamento, além de baixa resolutividade do caso; e consequências para os profissionais, como insegurança, desenvolvimento de resiliência, adoecimento, insatisfação, desestímulo e perda da credibilidade.

[…] *as questões negativas, elas são muito fortes nessa questão, que a gente carrega, o que a gente vai carregar para o resto da vida, mas eu também acredito firmemente que são nessas situações que a gente desenvolve resiliência, e são nessas situações que a gente aprende.* (GF1; E4)

*Realmente, eu concordo com a E2. Eu acho que a perda do vínculo é a maior consequência para nós enquanto UBS, porque fica muito mais difícil depois acompanhar todo o tratamento, reverter esse quadro* […] (GF5; E5)

Os resultados permitiram a construção do mapa conceitual das questões bioéticas presentes na prática de cuidado dos enfermeiros da ESF, soluções, facilitadores, dificultadores e consequências (Figura 2).

**Figura 2:** Mapa conceitual sobre as questões bioéticas do cuidado do enfermeiro na Estratégia Saúde da Família, São Paulo, Brasil, 2021

## DISCUSSÃO

Nesta pesquisa, identificou-se que a ESF é permeada por questões bioéticas que remetem à complexidade do cuidado nesse contexto devido à intensidade das relações, trabalho em equipe, pluralidade de valores imbricados no cuidado individual e familiar. Tais questões representam também a diversidade nos aspectos relacionados à bioética, conforme ilustrado no mapa conceitual.

Dentre as questões bioéticas identificadas nos grupos focais, as relacionadas ao trabalho em equipe, sigilo e privacidade e decorrentes da relação com usuários e famílias corroboram os achados de Zoboli e Fortes ^(4)^. No presente estudo, elas representaram 58% dos extratos, demonstrando sua relevância na atuação da ESF. Destaca-se que problemas éticos decorrentes de relações conflituosas dentro da equipe ou entre a equipe e usuários podem ser compreendidos na perspectiva da bioética do cuidado, cujo enfoque é na responsabilidade nas relações humanas.

Na concepção da bioética do cuidado de Gilligan, um problema ético emerge quando há conflito de responsabilidade nas relações humanas, e a resolução desse conflito visa garantir a manutenção dos relacionamentos de cuidado ^(14)^. Sob tal perspectiva, a solução para os problemas éticos dessa natureza, segundo Zoboli ^(15)^, consiste em acionar a rede de relações pela comunicação não violenta, cooperativa e não competitiva, que promova a inclusão de todos a fim de fortalecer em vez de romper as conexões existentes ^(15)^. Isso se demonstrou justamente nos relatos em que o vínculo e a continuidade terapêutica eram considerados importantes para os profissionais.

Quanto às questões bioéticas relacionadas ao sigilo e privacidade do usuário, embora presentes desde o principialismo, os resultados demonstraram que ainda ocorrem na prática de cuidado dos enfermeiros da ESF. Trata-se de aspecto inerente à prática da enfermagem, previsto no Código de Ética dos Profissionais de Enfermagem ^(11)^.

Segundo Junges e colaboradores ^(16)^, essa abordagem legal para resolução dos problemas de sigilo e confidencialidade da prática assistencial mostra-se insuficiente para responder às diversas situações requeridas durante o trabalho da ESF de cuidado longitudinal, o qual é característico do modelo de atenção que propõe abordagem de acolhimento, confiança e vínculo.

Os participantes consideraram que as questões bioéticas ligadas aos conflitos de valores eram provenientes da diversidade socioeconômica e cultural no contexto de atuação, e elas se configuram em conflitos quando contrastam com os valores morais dos profissionais. A literatura evidencia que a ocorrência de questões bioéticas no cuidado em saúde torna explícita, muitas vezes, as diferenças culturais, de valores e de crenças, de forma que as ações de cuidado podem desdobrar se em diferentes interpretações dos modos de viver ^(17)^.

No tocante aos dificultadores dos problemas bioéticos, os participantes elencaram as dificuldades no desenvolvimento de trabalho em equipe, essencialmente na sua efetivação, fragilidades da competência de trabalho em equipe, além da orientação do trabalho para cumprimento de metas e produtividade. Interferindo na assistência oferecida à comunidade, dificuldades relacionadas ao trabalho em equipe na ESF vêm sendo relatadas como obstáculo para o trabalho integrado: falta de cooperação entre profissionais, estruturas organizacionais rigidamente hierarquizadas, desigualdade técnica e social, alta rotatividade, quantitativo de profissionais insuficiente e baixa qualificação ^(18)^.

O caráter multiprofissional do trabalho das ESFs revela uma necessidade indispensável e, ao mesmo tempo, um desafio referente à transição da configuração de agrupamento de pessoas para a constituição de equipe, caracterizada pela interação e integração entre profissionais que resultam na constituição de um projeto comum ^(19)^. Ainda, embora não ressaltado pelos profissionais nos GFs, a estratégia de deliberação moral - assim como o trabalho em equipe - enquanto uma possibilidade de resolver os problemas bioéticos requer ação integrada e reconhece a limitação do cuidado e tomada de decisão individual ^(17)^.

Ratificando a literatura, os resultados dos grupos focais também mostraram que a gestão do serviço baseada essencialmente em indicadores de resultados foi um dificultador: trata-se de um dos principais fatores que atravessam o processo de trabalho da equipe e acarretam prejuízos na condução das questões bioéticas ^(19)^. Em outras palavras, no GF, os enfermeiros RTs reconheceram na qualidade de problemas bioéticos o fato de as metas de produtividade nem sempre se traduzirem em valores como acesso, qualidade e segurança do cuidado.

Além disso, elementos do próprio cotidiano do trabalho que demandariam ações da gestão do serviço revelam desgaste do trabalhador: sobrecarga de atividades, excesso de demanda, acúmulo de funções além das assistenciais, complexidade das demandas de saúde e dificuldades em atender as expectativas dos usuários. Isso repercute negativamente na garantia do acesso, na integralidade, na qualidade e segurança do cuidado ^(20-21)^.

Mesmo com as dificuldades apresentadas, os participantes apontaram enquanto soluções, para o enfrentamento das questões bioéticas, diferentes estratégias de tomada de decisão balizadas pelos valores dos princípios e diretrizes desse modelo de atenção, como integralidade, resolutividade e longitudinalidade do cuidado. Nesse sentido, observa-se a importância de considerar que o tema polissêmico da integralidade da atenção, “integralidade focalizada”, é resultante da integração dos vários saberes do cuidado multiprofissional; e “integralidade ampliada”, derivada da articulação de cada serviço da Rede de Atenção à Saúde além de instituições intersetoriais ^(22)^.

Outras soluções que eles propuseram para as questões bioéticas foram “conscientização e empatia” e “capacitação da equipe”. A primeira começa com a problematização dessas questões, visando garantir o direito constitucional à saúde e a assistência humanizada. Quanto à capacitação da equipe, para produzir mudanças nas práticas de cuidado, destacaram a necessidade de estabelecer diálogo sobre o processo de trabalho problematizando-o ^(23-24)^.

As soluções propostas nos GFs aproximam-se das propostas da bioética do cuidado, pois pressupõem resultados práticos que necessitam de relações dialógicas não coercitivas e mudança pela conscientização entre os envolvidos na questão bioética. Nesse contexto, a deliberação moral pode destacar-se não somente como método, mas também processo contínuo de aprendizagem da ética aplicada, baseada na construção coletiva de soluções práticas para problemas bioéticos, caracterizadas pelo exercício contínuo de averiguação de cursos intermediários e prudentes de decisão ^(17,25)^.

Os participantes reconheceram que a tomada de decisões em equipe e a elaboração de projetos terapêuticos singulares contribuíram para a solução mais assertiva e prudente diante das questões bioéticas identificadas. Tais escolhas relacionam-se aos cursos intermediários de ação e à abordagem problematizadora. Além disso, ao compartilhar o caso e decisão, também se observa o delineamento de uma responsabilidade comum à equipe e, consequentemente, diminuição da sobrecarga de trabalho e dos sofrimentos que possam decorrer dessas decisões.

A categoria de facilitadores contemplou o trabalho em equipe, uso de tecnologias leves e leve-duras de cuidado, apoio da RAS e educação permanente, matriciamento e a experiência profissional na área de atuação da AB. O “trabalho em equipe” foi percebido como facilitador quando presente e dificultador quando ausente. Quando o trabalho em equipe se efetiva no processo de trabalho, ele foi considerado como potente facilitador para a condução de questões bioéticas.

Ainda, observou-se o reconhecimento da importância do vínculo e da escuta qualificada enquanto propulsores para o cuidado integral e humanizado; e como facilitadores na condução de questões bioéticas na ESF para compreensão da concepção ampliada de saúde. O vínculo, na qualidade de tecnologia leve nas relações dentro da ESF, está inserido nas diretrizes basilares desse modelo de atenção, que estabelece a responsabilização pela área adstrita do território e, consequentemente, a necessidade de uma interação geradora de vínculos - de forma longitudinal, humanizada e integral - entre os trabalhadores da saúde e os usuários ^(26)^.

O apoio da Rede de Atenção à Saúde e a educação permanente também estiveram presentes tanto sendo facilitadores quanto soluções para manejar questões bioéticas. A ESF como estratégia de reorganização do sistema de saúde implica ser reconhecida enquanto coordenadora do cuidado e elemento central na comunicação com a RAS. A organização dos serviços de saúde em redes pressupõe mudança dos sistemas fragmentados e se efetiva por meio de uma Atenção Primária de qualidade, com equipes capazes de ampliar a ação interprofissional para além do âmbito local, envolvendo outras equipes de outros pontos da rede que atuam em parceria com usuários e comunidade, para alcançar a integralidade do cuidado ^(19)^.

Nesse sentido, os relatos dos GFs destacaram a relevância do matriciamento realizado pela equipe multiprofissional. Para eles, é uma potente ação de educação permanente em serviço, que contribui para a ampliação da clínica do enfermeiro e para a transposição do modelo biomédico. O processo da educação permanente para a temática da bioética inclui a problematização das situações vividas pelas equipes da ESF e mostra-se uma poderosa estratégia de qualificação para identificar e propor soluções a problemas éticos, incorporando conceitos, teorias e métodos da bioética ^(27)^.

Entre as principais consequências das questões bioéticas mencionadas pelos participantes dos GFs, destacaram-se: “consequências para os enfermeiros da ESF” e “consequências para usuários e família”. Para os enfermeiros, incluíram aspectos positivos (p.ex., desenvolvimento de resiliência para o enfrentamento), contudo consideraram negativa a maioria das consequências (insegurança, adoecimento, insatisfação, desestímulo e perda da credibilidade).

Estudo evidenciou que questões bioéticas no contexto da Atenção Primária afetam trabalhadores, usuários, gestores e a própria estrutura do trabalho em saúde. Assim, mostrou ser necessário que os profissionais da ESF fomentem discussões para melhorar a comunicação e o trabalho em equipe, bem como aprimorar habilidades fundamentais à prática interdisciplinar e ampliada que a ética na atenção à saúde pressupõe ^(28)^.

Neste estudo, a perda do vínculo e a crise familiar foram consequências identificadas para usuários e famílias no manejo da ocorrência das questões bioéticas que feriram o sigilo e a privacidade do usuário, seja na abordagem dilemática, seja na perspectiva legal, deontológica. A literatura também aponta o descrédito da equipe da ESF decorrente da quebra de confiança, enquanto indica que a comunicação é uma chave para sua solução ^(29)^.

A perspectiva legal focaliza os deveres expressos no código profissional, por vezes insuficientes para as questões bioéticas do cotidiano da ESF, as quais pressupõem práticas como acolhimento, confiança e vínculo; estes são necessários para pactuação do compartilhamento das informações entre usuários, família e profissionais, ao considerar o bem-estar, direitos do usuário e a intersubjetividade na discussão de problemas ^(16)^. De fato, na atuação da ESF, evidenciam-se questões bioéticas muito próprias, que deverão ser superadas mediante uma construção permeada por diálogo, com a participação de todos ^(24)^.

### Limitações do Estudo

As limitações deste estudo correspondem à necessidade de incluir a percepção de outros atores sobre as questões bioéticas na prática de cuidado do enfermeiro da ESF, como os usuários e os gestores dos serviços de saúde, para complementar os resultados encontrados. Vale destacar que, em decorrência do cenário epidemiológico relacionado à pandemia de covid-19, foi preciso mudar o formato de coleta dos dados, de presencial para virtual.

### Contribuições para a enfermagem e para o campo da saúde coletiva

O presente estudo contribui com estes elementos necessários: reflexão sobre as questões bioéticas inerentes à prática de cuidado e ao processo de trabalho dos enfermeiros na ESF; e reconhecimento da importância de utilizar os pressupostos da deliberação moral para a análise e resolução dessas questões.

## CONSIDERAÇÕES FINAIS

As questões bioéticas neste estudo remeteram a temáticas persistentes no campo da bioética e são um desafio, pois apresentam vários cursos de ação possíveis, logo demandam ponderação para escolha da melhor alternativa.

A compreensão das questões bioéticas envolvidas na gestão do cuidado dos enfermeiros que atuam na Estratégia Saúde da Família (ESF) foi possível por meio da utilização de diferentes correntes bioéticas como referencial teórico. Neste estudo, foram observadas aproximações com o principialismo, bioética do cuidado e correntes de interface da saúde coletiva: bioética da proteção e da intervenção. Destaca-se que, para a compreensão da complexidade das questões bioéticas nesse contexto de cuidado, torna-se necessário a complementaridade de diferentes correntes.

Foram percebidos mais dificultadores do que facilitadores na condução das questões bioéticas, o que remete à necessidade de reorganização do processo de trabalho das equipes, a fim de possibilitar a identificação, análise e proposição de soluções dessas questões.

As soluções para as questões bioéticas relacionaram-se às decisões coletivas, o que reforça a importância da equipe interprofissional e interdisciplinar na averiguação das questões bioéticas para alcance de cursos intermediários de ação. Tais soluções são alcançadas quando há cuidado longitudinal, educação permanente e abordagem sistematizada proposta pela deliberação moral.

Os facilitadores identificados nesta pesquisa convergiram fortemente para as soluções de questões bioéticas, tendo em vista a qualidade da assistência e a prevenção de consequências deletérias para os profissionais e usuários, família e comunidade.

As implicações e recomendações para a prática de cuidado das questões bioéticas incluem: promover educação permanente, com apoio de metodologias problematizadoras de aprendizagem; valorizar a singularidade do cuidado, uso de tecnologias leves; propiciar espaços de discussão, abordagem estruturada - por exemplo, a metodologia da deliberação moral para tomar decisões; investir em estratégias que fortaleçam o trabalho em equipe e contribuam para a resolutividade das necessidades, visando potencializar princípios e diretrizes do SUS; desenvolver indicadores qualitativos do cuidado, que reconheçam aspectos relacionais, nas ações realizadas na ESF, como humanização, empatia, vínculo, acolhimento, respeito à autonomia, clínica ampliada e satisfação dos usuários nos resultados assistenciais.

As implicações para pesquisas incluem a elaboração de estudos que compreendam a percepção dos demais atores, usuários e gestores, a fim de ampliar o conhecimento sobre questões bioéticas e suas abordagens na gestão do cuidado na ESF, com destaque para metodologias que abranjam a problematização, por exemplo, grupo focal e pesquisa-ação.
